# An Emerging Infectious Disease Triggering Large-Scale Hyperpredation

**DOI:** 10.1371/journal.pone.0002307

**Published:** 2008-06-04

**Authors:** Marcos Moleón, Pablo Almaraz, José A. Sánchez-Zapata

**Affiliations:** 1 Departamento de Biología Animal, Universidad de Granada, Granada, Spain; 2 Estación Biológica de Doñana, Consejo Superior de Investigaciones Científicas, Sevilla, Spain; 3 Departamento de Biología Aplicada, Universidad Miguel Hernández, Orihuela, Alicante, Spain; University of California, Berkeley, United States of America

## Abstract

Hyperpredation refers to an enhanced predation pressure on a secondary prey due to either an increase in the abundance of a predator population or a sudden drop in the abundance of the main prey. This scarcely documented mechanism has been previously studied in scenarios in which the introduction of a feral prey caused overexploitation of native prey. Here we provide evidence of a previously unreported link between Emergent Infectious Diseases (EIDs) and hyperpredation on a predator-prey community. We show how a viral outbreak caused the population collapse of a host prey at a large spatial scale, which subsequently promoted higher-than-normal predation intensity on a second prey from shared predators. Thus, the disease left a population dynamic fingerprint both in the primary host prey, through direct mortality from the disease, and indirectly in the secondary prey, through hyperpredation. This resulted in synchronized prey population dynamics at a large spatio-temporal scale. We therefore provide evidence for a novel mechanism by which EIDs can disrupt a predator-prey interaction from the individual behavior to the population dynamics. This mechanism can pose a further threat to biodiversity through the human-aided disruption of ecological interactions at large spatial and temporal scales.

## Introduction

Pathogens can exert a large influence on ecological interactions from the individual to the ecosystem level [1]. In particular, Emerging Infectious Diseases (EIDs) are currently regarded as one of the major threats to wildlife and provide an outstanding paradigm of the interaction between humans and biodiversity [2], [3]. Although the realm of community interactions that can be impacted upon by pathogens is strikingly diverse [1], we are just beginning to understand the role of EIDs on wildlife dynamics at large temporal and spatial scales [4].

In 1988 a major viral outbreak of Rabbit Hemorrhagic Disease (RHD) rapidly spread throughout Spain [5], [6]. The RHD is highly virulent and affects both adults and young of European rabbit *Oryctolagus cuniculus*, mainly during the breeding period [6]. The rate of spatial spread of the disease was strikingly high after the initial outbreak, so human intervention through the translocation of infected individuals for hunting purposes is highly likely [5]. Within a short time period the epidemic caused many local extinctions and fragmentations in previously large populations [5]–[7], with substantial economic and ecosystem consequences [e.g., 6]. Due to this rapid increase in prevalence and geographic range of the disease, RHD can be considered an Emerging Infectious Diseases (EIDs) [8], a group of diseases usually facilitated by humans [2], [3], [8].

Here we compile long-term hunting bag records for the European rabbit and the red-legged partridge *Alectoris rufa* in continental Spain. Both are the two most important Spanish small game quarry species and the main prey for a diverse community of predators, including some critically endangered species [9]. Due to similarity in size, habitat preferences and ground-related behaviour, the partridge is potentially an alternative prey for shared predators when rabbits become scarce [10]. Indeed, available evidence suggest a striking change in the relative composition of both prey species in the diet of some of some of the major predators in Spain due to the RHD outbreak [e.g., 11]. Thus, our goal in this paper is to assess if the RHD outbreak left a dynamic fingerprint both in the rabbit population, through direct mortality from the disease, and in the partridge population, through hyperpredation induced by an increased predation pressure from shared predators.

### The EID-mediated hyperpredation hypothesis

In a broad sense, hyperpredation can be defined as an enhanced predation pressure on a secondary prey due to either: a) an increase in the abundance of the predator population caused by an abrupt increase in the abundance of its main prey; or b) a sudden drop in the abundance of the main prey [12], [13]. Theoretical evidence suggest that this enhanced predation, which does not necessarily mean very high, but rather ‘higher-than-normal’ levels of consumption, could be enough to result in demographic effects in prey populations [13], with the only condition of predation being additive to other mortality causes. To date the available empirical evidence on hyperpredation is restricted to cases in which the introduction of a feral prey caused overexploitation of native prey [e.g.], [ 12]–[17]. Based on the population dynamics theory of infectious diseases, and on the modelled effects of the RHD on the demography of the European rabbit [18], we can predict three dynamic phases in the interaction between the RHD and the rabbit: 1) A pre-outbreak phase, with a large population density on average; 2) A post-outbreak phase during which a large portion of the individuals become infected and die, hence causing a population drift towards a low density period. The intensity and duration of this phase depends, among other things, on the rate of spatial spread of the disease; and 3) A post-immunization phase, in which large-scale mortality rates decrease due to the immunization of rabbits and cause a return towards medium-high population densities. Due to the large numbers of predatory species shared by the European rabbit and the red-legged partridge in Spain, we can hypothesize that the RHD can potentially impact indirectly on the dynamics of the latter through hyperpredation. The RHD outbreak could thus potentially cause synchronized dynamics in the abundance of the infected host and the hyperpredated species. In this paper we test this prediction using both feeding data of shared predators and population dynamics data of the prey species.

## Results

**Figure 1:**
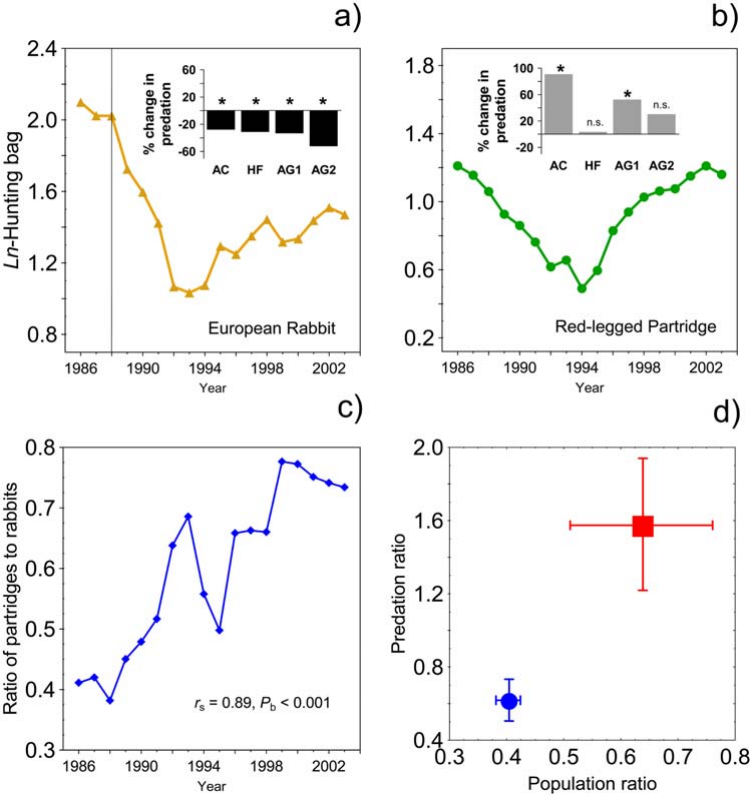
Time series of the log*_e_*-transformed hunting bag records for the European rabbit (a) and red-legged partridge (b) in continental Spain, corrected for hunting effort. The vertical line in the figure denotes the temporal location of the RHD outbreak. Within each graph, the small figure shows the change in predation after the RHD outbreak, defined as the percentage shift in the relative abundance of each species in the diet of three major shared predators (see Table S1). * p<0.05; n.s. = non-significant (according to references given in Table S2). AC represents the golden eagle *Aquila chrysaetos*; HF, Bonelli's eagle *Hieraaetus fasciatus*; AG1, northern goshawk *Accipiter gentilis* in scarcely forested areas; AG2, northern goshawk in heavily forested areas; c) Temporal evolution of the ratio of partridge to rabbits hunted in continental Spain (*r*_s_ = Spearman's rank order correlation; *P*_b_ = bootstrapped *P*-value, constructed using 1000 samples); d) The average population ratio of partridges to rabbits during the pre-outbreak (blue circles) and post-outbreak phases (red squares), is plotted against the average ratio of partridges to rabbits in the diet of predators prior and after the outbreak. This latter ratio was weighted by the absolute proportion of partridges and rabbits in the diet of each predator.

The population trajectory of both prey species is shown in Figure 1a and b. A severe drop in abundance is evident after the RHD outbreak in the rabbit, but the decline is evident as well in the partridge. From the mid-nineties, a recovery period is observed in both species. However, this recovery seems more evident in the time series of the partridge, since the ratio of partridge to rabbit rises through time and nearly doubles at the end of the series (Fig. 1c). We compared the relative contribution of partridge and rabbit in the diet of their three major shared predators throughout Spain (Table S1) before and after the RHD outbreak. The rabbit was the primary prey for most of these predators (Table S2). Significant decreases in the relative composition of rabbit in the diet of all predators were found after the outbreak; in contrast, the relative composition of partridge increased significantly after the outbreak in most cases (Figure 1; Table S2), which provides evidence for enhanced predation pressure. There is indeed a close correspondence between both the population ratio and the predation ratio of partridge to rabbits before and after the RHD outbreak (Fig. 1d).

**Figure 2:**
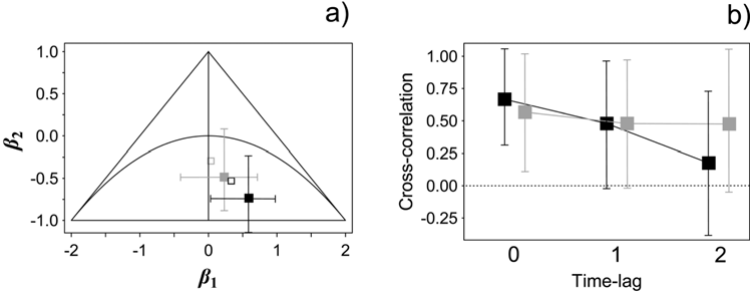
a) Royama diagram [19], showing the dynamics of the second-order model in the parameter space. Outside the triangle, populations tend to extinction, and below the parabola multiannual cycles arise; within the area between the triangle and the parabola the system exhibit dampened stability (on the right) or two-years cycles (on the left). The grey and black solid square depict the parameter combination for the European rabbit and red-legged partridge model, respectively. Open squares denote the parameter combination for the same model but without the state-space component (*τ* ^2^ = 0). b) Cross-species correlation coefficients between the hidden states time series (black squares) and residuals time series (grey squares) at 0, 1 and 2 time lags. Both the states and residuals time-series are average estimates obtained through model averaging (see [33]). The whiskers stand for the 95% confidence interval of each coefficient, constructed using bootstrapping (1,000 samples).

Table 1 shows the posterior estimates for the parameters in the set of state-space models fitted to the European rabbit and red-legged partridge time-series. The relative effect of sampling variability is low compared to process error in both species. According to the BIC, a model with direct density-dependence is selected in the European rabbit population and a second-order model is selected for the red-legged partridge; the behaviour of the MCMC simulation approach is very good in both models (Figure S1). Interestingly, the effect of rainfall fluctuations is negligible in both prey populations. The Royama diagram [19] suggests that the asymptotic behaviour of each time-series is consistent with multiannual-cycles (Figure 2a). Using the state estimates averaged across models, we found evidence of direct cross-species correlation (Figure 2b). This suggests that the dynamics of both prey species were synchronized at a large-spatio-temporal scale, with no time-lag.

**Table 1:** Posterior estimates for the parameters in the Gompertz state-space model (eqns. 1–3), obtained for both prey species through Markov chain Monte Carlo integration.

Species & model*	*β*_0_ (±SE)	*β*_1_ (±SE)	*β*_2_ (±SE)	*γ* (±SE)	*σ* ^2^ (±SE)	*τ* ^2^ (±SE)	BIC¶
**European rabbit**							
**X** ***_i_*** ** [** ***β*** **_0_ · · ·]**	−0.035 (0.039)	—	—	—	0.025 (0.011)	0.003 (0.004)	41.625
**X** ***_i_*** ** [** ***β*** **_0_ · · ** ***γ*** **]**	1.055 (0.937)	—	—	−0.187 (0.160)	0.023 (0.013)	0.004 (0.004)	44.927
**X** ***_i_*** ** [** ***β*** **_0_** ***β*** **_1_ · ·]**	0.313 (0.164)	**−0.239 (0.110)**	—	—	0.019 (0.011)	0.004 (0.004)	**32.496**
**X** ***_i_*** ** [** ***β*** **_0_** ***β*** **_1_ · ** ***γ*** **]**	−0.230 (0.853)	**−0.265 (0.121)**	—	0.099 (0.154)	0.020 (0.011)	0.004 (0.005)	35.822
**X** ***_i_*** ** [** ***β*** **_0_** ***β*** **_1_** ***β*** **_2_ ·]**	**0.342 (0.134)**	0.232 (0.295)	−0.486 (0.255)	—	0.010 (0.009)	0.004 (0.004)	33.261
**X** ***_i_*** ** [** ***β*** **_0_** ***β*** **_1_** ***β*** **_2_** ***γ*** **]**	−0.266 (0.775)	0.143 (0.301)	−0.456 (0.246)	0.118 (0.145)	0.012 (0.009)	0.004 (0.005)	33.283
**Red-legged partridge**							
**X** ***_i_*** ** [** ***β*** **_0_ · · ·]**	0.000 (0.030)	—	—	—	0.014 (0.006)	0.002 (0.002)	41.651
**X** ***_i_*** ** [** ***β*** **_0_ · · ** ***γ*** **]**	0.736 (0.698)	—	—	−0.126 (0.120)	0.014 (0.006)	0.002 (0.002)	44.720
**X** ***_i_*** ** [** ***β*** **_0_** ***β*** **_1_ · ·]**	0.127 (0.131)	−0.140 (0.141)	—	—	0.014 (0.007)	0.002 (0.002)	31.936
**X** ***_i_*** ** [** ***β*** **_0_** ***β*** **_1_ · ** ***γ*** **]**	0.169 (0.762)	−0.138 (0.157)	—	−0.008 (0.135)	0.016 (0.009)	0.002 (0.002)	35.822
**X** ***_i_*** ** [** ***β*** **_0_** ***β*** **_1_** ***β*** **_2_ ·]**	0.157 (0.090)	**0.591 (0.246)**	**−0.736 (0.236)**	—	0.005 (0.005)	0.002 (0.002)	**30.406**
**X** ***_i_*** ** [** ***β*** **_0_** ***β*** **_1_** ***β*** **_2_** ***γ*** **]**	0.527 (0.530)	**0.622 (0.253)**	**−0.736 (0.239)**	−0.068 (0.095)	0.005 (0.005)	0.003 (0.002)	33.875

* The range of possible models within the saturated one is ordered for each species according to increasing complexity. The modelled population process, denoted by the state variable **X** ***_i_***, can be affected by a range of parameters: *β*_0_ stands for the density-independent growth rate; *β*_1_ stands for first-order density-dependence; *β*_2_ denotes second-order density-dependence; *γ* stands for the weather effect on population size; and *σ* ^2^, *τ* ^2^ stand for the process and sampling variances, respectively. Shown are the mean ± Standard Error. Parameters in which the 95% credible interval does not overlap 0 are shown in bold type.

¶ Bayesian Information Criterion; the model minimizing this quantity is selected as the best descriptor of the dataset within the pool of fitted models, and is shown in bold type.

## Discussion

**Figure 3:**
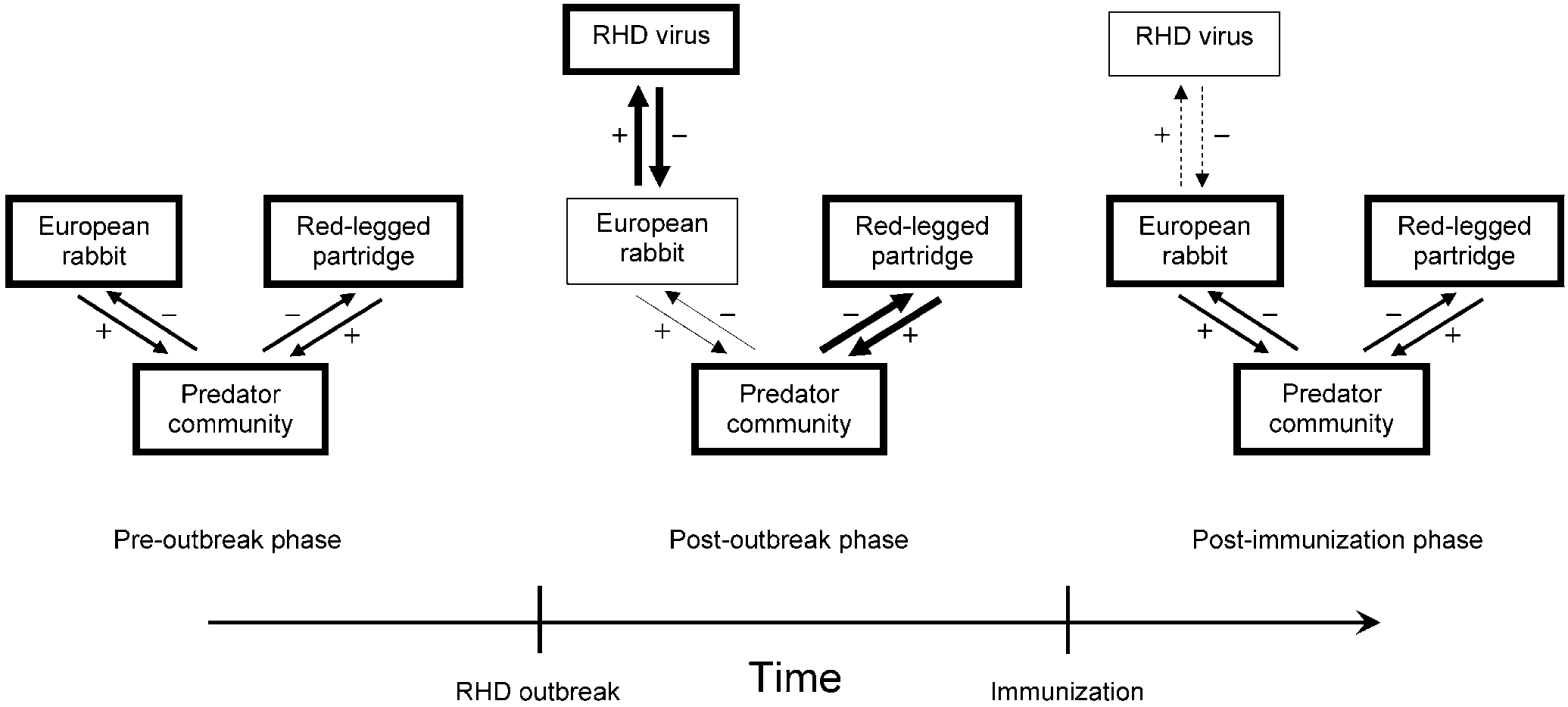
Graphical model depicting the ‘EID-mediated hyperpredation hypothesis’. The thickness of each arrow denotes the numerical intensity of the interaction, while the thickness of each box represents the relative contribution of each species/community to the intensity of the multi-species interaction. Before the outbreak of the Rabbit Hemorrhagic Disease (RHD), the predators community fed differentially upon both prey species according to “average” traits (individual preferences, life-history, etc.) and environmental factors (local prey availability, etc.). At the onset of the RHD outbreak, the abundance of the host species, which is the main prey, drops abruptly, so the intensity of predation shifts towards the secondary prey. This causes its population abundance to decrease accordingly. Once the proportion of immunized hosts begins to increase, the intensity of the inter-specific interaction returns to the “average” (pre-outbreak) levels. The population sizes of both prey species begin to rise as well at this stage. However, a proportion of RHD viruses can prevail in the host population.

To the best of our knowledge, we have provided the first evidence of an enhanced predation pressure on a secondary prey due to the population collapse of a primary prey induced by an EID. After a RHD outbreak, the reduced abundance of rabbits might force predators to focus on the partridge populations, therefore causing a numeric reduction in its population as well. Empirical evidence suggests that the prevalence of acquired immunity through antibodies to RHD increased during the mid-1990s in at least some local rabbit populations [6]. The immunization of rabbits with antibodies to the RHD and the subsequent numeric recovery of their populations should release partridge populations from enhanced predatory pressure (Figure 3). A shifting pattern is strikingly apparent after this period in both species, which suggests a major role for large-scale host immunization in generating the shifting pattern.

Although a specific prediction of the EID-mediated hyperpredation hypothesis is a significant cross-species correlation in the abundance of both prey, we recognize that some external factors, such as climate and habitat conditions, could act to synchronize their population dynamics. Nevertheless, these factors should have no obvious impact on the feeding behavior of major predators, which conspicuously changed after the RHD outbreak, and we have already shown that climate seems to have no effect on the dynamics of neither prey population. In addition, the structure and functioning of Mediterranean ecosystems are strongly dependent on rainfall fluctuations [20], so it is unlikely that habitat changes can be claimed as a putative factor globally explaining cross-species synchrony.

Besides hyperpredation, other factors might have acted to reduce partridge populations. Specifically, habitat destruction and deterioration, over-hunting and the use of pesticides have been claimed as factors triggering declines of local partridge populations, but it is also known that other local populations increased at the same time [21]. In particular, the artificial release of partridges into the wild has been a standard management procedure in Spain from the mid-eighties, noticeably increasing in magnitude and spatial coverage during recent years [21]. However, the continental partridge population analyzed in the present study only began to rise during the mid-nineties, and we believe that a major proportion of the partridges hunted from this period onwards is composed of partridges released from captive breeding. Since nearly all of the released partridges are known to be hunted by humans, we believe that the most recent hunting bag estimates are artificially high with respect to the natural density of partridges. Therefore, our estimates of cross-species correlation are likely conservative.

Another possibility is that during the low-abundance period of both prey some predators responded numerically by reducing their populations as well; this could have released both prey populations from predation and further enhance synchrony. However, we emphasize that this possibility is not incompatible with the EID-mediated hyperpredation. Moreover, there is no global evidence that the predator populations responded numerically during this period, and the available evidence suggests in fact that many local populations of the raptors considered in the present study increased due to efficient conservation measures [21].

Overall, using data from two key game species, we have provided evidence for a novel pathway by which EIDs can disrupt a predator-prey interaction from the individual behavior to the population dynamics. Due to multiple introductions and/or translocations of pathogens by humans, one of the major current threats of EIDs is an extremely fast and unpredictable rate of spatial spread of the disease [2]. Our analysis suggests that EID-induced hyperpredation can pose a further threat to wildlife through the human-aided disruption of ecological interactions at large spatial and temporal scales. This provides an example of the previously underappreciated impacts that an invasive disease can have on species other than the primary host.

## Materials and Methods

### Data set

To model the population dynamics of the European rabbit and the red-legged partridge, we gathered hunting bag data from the Spanish Ministry of Agriculture, Fisheries and Food. Available data span the 50 Spanish provinces from 1986 to 2003. However, the rabbit is native only to the 45 continental provinces, so we used data for those areas. Because hunting effort can influence the number of rabbits and partridges hunted [22], [23], we divided hunting bag data by the number of hunting licenses per year to obtain a reliable estimate of population size [7], [23].

To asses the numeric role of rabbits and partridges as prey for the Iberian predators (raptors and carnivorous mammals), we used our own field data and also conducted a bibliographic review of published data on feeding ecology of their predators (References S1). Egg predation was not considered in our review. Any predator including more than 5% of relative frequency of rabbit/partridge in its diet was considered as a rabbit/partridge consumer. We then examined whether rabbit consumers and partridge consumers were shared (Table S1). Overall, we gathered spatially segregated information on comparative diet of the main rabbit and partridge predators both before (in the “high prey density phase”) and after (in a “low prey density phase”) the RHD outbreak (Table S2). This allowed us to assess the impact of the viral outbreak in the relative diet composition of the main shared predators.

### Population dynamics modeling and cross-species correlation

In order to test for cross-species correlation in the population dynamics, we fitted a log-linear (Gompertz) model of population growth to the abundance of each species [24]. Recent evidence suggests a pervasive effect of rainfall on the population dynamics of a bird species at a national scale in Spain during the same study period [25]. Thus, we gathered information on the monthly variations in precipitation throughout the study area [25] and extended the simple Gompertz model by including rainfall as an environmental covariate. We used a state-space approach to control for sampling variability during parameter estimation [24], [26]. Be ***X*** ^T^ = [*X*_1_, *X*_2_, …, *X*_T_] the vector of the time series for the hidden (unobserved or latent) log-transformed abundances, *X*_i_. These are the states of the true population system. Be ***r*** ^T^ = [*r*_1_, *r*_2_, … , *r*_ T_] the vector of the time series for the amount of rainfall during each year (measured from December to December). The state equation for the temporal evolution of this sequence can be written as
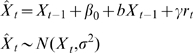
(1)were 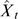 is the one-step ahead predicted population size, which is drawn from a normal distribution with the hidden population size at time *t*, *X_t_*, as the mean, and process variance σ^2^. Through a simple factorization, eqn. (1) can be simplified to
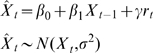
(2)where *β*_1_ = (1+*b*) is the coefficient for first-order density dependence [24], *γ* is a parameter measuring the effect of rainfall on population size, and *β*_0_, also known as the drift parameter, is a constant measuring the density- and environmental-independent growth rate (that is, when *β*_1_ = 1, *γ* = 0). Since the hidden abundances are, by definition, unobserved, we must link them to the observations through an observation model:
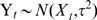
(3)were *Y_t_* is the log-transformed hunting bag record at time *t* and τ^2^ is the sampling variance. Both eqns. (2) and (3) are called the Gompertz state-space population abundance model [24], extended through the inclusion of an environmental covariate. Since the numeric interaction between predator and prey populations can involve several time-lags [27], we tested for delayed density-dependence. Therefore, the final model becomes:
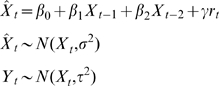
(4)were *β*_2_ is the coefficient for second-order density dependence. Solving linear state-space models through Maximum Likelihood techniques is feasible, but the estimation process can yield an extremely complex likelihood profile and the convergence of parameters and states cannot always be guaranteed [24]. Here we adopt a Bayesian approach and estimate parameters and states through Markov Chain Monte Carlo (MCMC, [28], [29]). This approach uses an iterative simulation scheme, so that the model need not be resolved analytically (see [30] for an introduction to MCMC techniques in ecology). A Bayesian model consists of a likelihood (or data) model, including the information contained in the data, and a prior (or parameter) model, containing previous information on parameter values. A posterior distribution for both parameters and states is obtained through the confrontation of both models, following the Bayes theorem [28]. We fitted the saturated model in eqn. 4 and a family of alternative models constructed by dropping some parameters from the saturated one: 1) a random walk with drift (no density-dependence, no climate effect); 2) a density-independent model with climatic effect; 3) a direct density-dependent model (AR1 model; [19]); 4) a direct density-dependence model with climatic effect; 5) a second-order density-dependence model; and 6) a second-order density-dependence model with climate effect. We compared the relative performance of each model using the Bayesian Information Criterion (BIC; [31]), with the model minimizing this quantity selected as the best descriptor of the dataset. A difference in BIC >1 was considered sufficient evidence in favour of the model with a smaller BIC. Since we had no previous information on parameter values, we constructed a prior model with vague distributions [28]. Values for the scale parameters were drawn from extremely flat (platikurtic) normal distributions: *β*_0_, *β*_1_, *β*_2_, *γ*∼*N*(0, 10^6^), while the variance parameters were given inverse-gamma distributions: *σ* ^2^, *τ* ^2^∼*IG*(10^−3^, 10^3^). Through this approach, we let the likelihood to dominate the prior in the posterior distribution. We initiated the MCMC simulation scheme for each model using three chains with different sets of initial values, and ran each chain for 300,000 iterations. We discarded the first 100,000 samples as a burn-in period, and used the remaining 200,000 to construct the posterior distributions of parameters and states. We used standard diagnostic tests for MCMC simulation [32] to check that the Markov chains were both stationary and uncorrelated, and to asses that the posterior density kernels conformed to the underlying statistical distributions.

We tested for cross-species correlation in the population dynamics of the partridge and rabbit populations using the point estimates for the hidden states in ***X*** ^T^. We used model averaging to obtain a final estimate of the state evolution, constructed by weighting each point estimate by the individual weight of each fitted model (see [33]); however, we emphasize that the correlation in the state estimates across models is very high (*r*>0.9), so the results are consistent among models. To test for time lags we performed the following analyses with the hidden time series of the red-legged partridge lagged up to two years with respect to the rabbit. Two different analyses were performed. First, we correlated directly the estimates in ***X*** ^T^ for both species. This yielded estimates of cross-correlation while controlling for both sampling and process error, but not density-dependence and climate effects. Second, we used the residuals of the state equation in (4), 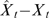. This provided estimates of cross-species correlation while controlling for sampling error, process error, density-dependence and climate. In either case, a positive cross-correlation coefficient will indicate that the population abundances of the prey species are fluctuating synchronously through time.

## Supporting Information

Figure S1 The Monte Carlo trace of the three Markov Chains (shown in green, red and blue) is shown for each parameter in the state-space model selected as the most parsimonious by the BIC, for both the European rabbit (a) and the red-legged partridge (c). Each chain was initiated with slightly different parameter values. Also shown are the probability density kernels of each parameter, constructed after joining the three chains. The autocorrelation plot is shown for each chain after thinning the MCMC samples every 20 iterations, for both the European rabbit (b) and the red-legged partridge (d). Finally, the Gelman-Rubin statistic calculates the rate of convergence of each chain across the MCMC simulation. A value near to 1 indicates a correct convergence of the chain. *β*_0_ stands for the density-independent growth rate; *β*_1_ stands for first-order density-dependence; *β*_2_ denotes second-order density-dependence; *γ* stands for the weather effect on population size; and *σ*_2_, *τ*_2_ stand for the process and sampling variances, respectively.
(13.43 MB TIF)

Table S1 (0.06 MB DOC)

Table S2 (0.05 MB DOC)

References S1 (0.04 MB DOC)

## References

[1] Hatcher MJ, Dick JTA, Dunn SD (2006). How parasites affect interactions between competitors and predators.. Ecol Lett.

[2] Daszak P, Cunningham AA, Hyatt AD (2000). Emerging infectious diseases of wildlife–Threats to biodiversity and human health.. Science.

[3] Anderson PK, Cunningham AA, Patel NG, Morales FJ, Epstein PR (2004). Emerging infectious diseases of plants: pathogen pollution, climate change and agrotechnology drivers.. Trends Ecol Evol.

[4] LaDeau SL, Kilpatrick AM, Marra PP (2007). West Nile virus emergence and large-scale declines of North American bird populations.. Nature.

[5] Villafuerte R, Calvete C, Blanco JC, Lucientes J (1995). Incidence of viral hemorrhagic disease in wild rabbit populations in Spain.. Mammalia.

[6] Calvete C, Estrada R, Villafuerte R, Osácar JJ, Lucientes J (2002). Epidemiology of viral hemorrhagic disease (VHD) and myxomatosis in the wild rabbit (*Oryctolagus cuniculus*) in the mid-Ebro valley, Spain.. Vet Rec.

[7] Virgós E, Cabezas-Díaz S, Lozano J Is the wild rabbit (*Oryctolagus cuniculus*) a threatened species in Spain? Sociological constraints in the conservation of species.. Biodivers Conserv. In press.

[8] Morse SS (1995). Factors in the emergence of infectious diseases.. Emerg Infect Dis.

[9] Moleón M, Barea-Azcón JM, Moleón M, Travesí R, Ballesteros-Duperón E, Luzón JM (2007). El estudio del impacto de los predadores sobre las presas cinegéticas: un intento de compatibilizar caza y conservación.. Biodiversidad y Conservación de Fauna y Flora en Ambientes Mediterráneos.

[10] Angelstam P, Lindström E, Widen P (1985). Synchronous short-term population fluctuations of some birds and mammals of Fennoscandia–occurrence and distribution.. Holarctic Ecol.

[11] Fernández C (1993). Effect of the viral haemorrhagic pneumonia of the wild rabbit on the diet and breeding success of the Golden Eagle *Aquila chrysaetos* (L.).. Rev Ecol Terre Vie.

[12] Smith AP, Quin DG (1996). Patterns and causes of extinction and decline in Australian conilunire rodents.. Biol Conserv.

[13] Courchamp F, Langlais M, Sugihara G (2000). Rabbits killing birds: modelling the hyperpredation process.. J Anim Ecol.

[14] Roemer GW, Coonan TJ, Garcelon DK, Bascompte J, Laughrin L (2001). Feral pigs facilitate hyperpredation by golden eagles and indirectly cause the decline of the island fox.. Anim Conserv.

[15] Kristan WB, Boarman WI (2003). Spatial patterns of risk of common raven predation on desert tortoises.. Ecology.

[16] Whelan CJ, Brown JS, Maina G (2003). Search biases, frequency-dependent predation and especies co-existence.. Evol Ecol Res.

[17] Sheppard SK, Bell J, Sunderland KD, Fenlon J, Skervin D (2005). Detection of secondary predation by PCR analyses of the gut contents of invertebrate generalist predators.. Mol Ecol.

[18] Calvete C (2006). Modeling the effect of population dynamics on the impact of rabbit haemorrhagic disease.. Conserv Biol.

[19] Royama T (1992). Analytical Population Dynamics..

[20] Blondel J, Aronson J (1999). Biology and wildlife of the Mediterranean region..

[21] Madroño A, González C, Atienza JC (2004). Libro Rojo de las Aves de España..

[22] Bostford LW, Methot RD, Johnston WE (1983). Effort dynamics of the northern California Dungeness crab (*Cancer magister*) fishery.. Can J Fish Aquat Sci.

[23] Cattadori IM, Haydon DT, Thirgood SJ, Hudson PJ (2003). Are indirect measures of abundance a useful index of population density? The case of red grouse harvesting.. Oikos.

[24] Dennis B, Ponciano JM, Lele SR, Taper ML, Staples DF (2006). Estimating density dependence, process noise, and observation error.. Ecol Monogr.

[25] Almaraz P, Amat JA (2004). Complex structural effects of two hemispheric climatic oscillators on the regional spatio-temporal expansion of a threatened bird.. Ecol Lett.

[26] Durbin B, Koopman SJ (2001). Time series analysis by state space methods..

[27] Turchin P (2003). Complex population dynamics. Monographs in Population Biology..

[28] Gelman A, Carlin JB, Stern HS, Rubin DB (2004). Bayesian data analysis. 2nd edition..

[29] Robert CP, Casella G (2005). Monte Carlo statistical methods. 2nd edition..

[30] Clark J (2007). Models for ecological data..

[31] Tong H (1990). Non-linear time series. A dynamical system approach..

[32] Brooks S, Gelman A (1998). General methods for monitoring convergence of iterative simulations.. J Comp Graph Stat,.

[33] Burnham KP, Anderson DR (1998). Model selection and inference: a practical information–theoretic approach..

