# Supplementary material for: An Emerging Infectious Disease Triggering Large-Scale Hyperpredation
Source: PLoS One. 2008 Jun 4;3(6):e2307. doi: 10.1371/journal.pone.0002307 (PMC2390756; doi:10.1371/journal.pone.0002307)
Supplement: Table S1 — (0.06 MB DOC) [file pone.0002307.s002.doc]

**Table S1**. Spanish predators including >5% of European rabbit (“rabbit consumers”) and red-legged partridge (“partridge consumers”) in their diet in at least one site or season. Shared predators are in boldface. Asterisks indicates predators consuming >20% of rabbits or partridges [1-11; own data]. Predators for which diet data were gathered before and after the RHD outbreak are shown in red.

| **Rabbit consumers**  **(47.9% of Spanish predators)** | **Partridge consumers**  **(18.8% of Spanish predators)** |
| --- | --- |
| ***Aquila chrysaetos* *** | ***Aquila chrysaetos* *** |
| ***Hieraaetus fasciatus* *** | ***Hieraaetus fasciatus* *** |
| ***Accipiter gentilis* *** | ***Accipiter gentilis* *** |
| ***Circus aeruginosus*** | ***Circus aeruginosus*** |
| ***Hieraaetus pennatus* *** | ***Hieraaetus pennatus*** |
| ***Buteo buteo* *** | ***Buteo buteo*** |
| ***Mustela nivalis*** | ***Mustela nivalis*** |
| ***Mustela putorius* *** | ***Mustela putorius*** |
| ***Lynx pardinus* *** | ***Lynx pardinus*** |
| *Milvus milvus* * |  |
| *Milvus migrans* * |  |
| *Aegypius monachus* * |  |
| *Neophron percnopterus* * |  |
| *Aquila adalberti* * |  |
| *Bubo bubo* * |  |
| *Strix aluco* * |  |
| *Canis lupus* * |  |
| *Vulpes vulpes* * |  |
| *Martes foina* |  |
| *Herpestes ichneumon* * |  |
| *Meles meles* * |  |
| *Genetta genetta* |  |
| *Felis silvestris* * |  |
