# Supplementary material for: An Emerging Infectious Disease Triggering Large-Scale Hyperpredation
Source: PLoS One. 2008 Jun 4;3(6):e2307. doi: 10.1371/journal.pone.0002307 (PMC2390756; doi:10.1371/journal.pone.0002307)
Supplement: Table S2 — (0.05 MB DOC) [file pone.0002307.s003.doc]

**Table S2**. Frequency of occurrence of European rabbit and red-legged partridge in the diet of the three main shared predators in Spain. The data belong to a 7-year period before and after the RHD outbreak. For each predator, diet comparisons were performed within the same study area: Navarra region for the golden eagle [2], northeastern quadrant of Spain for the Bonelli’s eagle [10], and Catalonia region for the northern goshawk [7]. (1): scarcely forested areas; (2): heavily forested areas.

|  | % of rabbit in diet | | % of partridge in diet | |
| --- | --- | --- | --- | --- |
| Predator | Before RHD | After RHD | Before RHD | After RHD |
| *Aquila chrysaetos* | 56.0 | 40.6 | 7.4 | 14.1 |
| *Hieraaetus fasciatus* | 31.1 | 21.5 | 13.4 | 13.8 |
| *Accipiter gentilis* (1) | 21.8 | 14.6 | 15.0 | 22.8 |
| *Accipiter gentilis* (2) | 10.0 | 4.8 | 18.6 | 24.1 |
