# Supplementary material for: An Emerging Infectious Disease Triggering Large-Scale Hyperpredation
Source: PLoS One. 2008 Jun 4;3(6):e2307. doi: 10.1371/journal.pone.0002307 (PMC2390756; doi:10.1371/journal.pone.0002307)
Supplement: References S1 — (0.04 MB DOC) [file pone.0002307.s004.doc]

**Supporting references**

1. Corbacho C, Morán R, Villegas A (2005) La alimentación del aguilucho cenizo *Circus pygargus* en relación a los usos del suelo en áreas pseudoestepáricas de Extremadura (SO Península Ibérica). Ardeola 52: 3-19.
2. Fernández C (1993) Effect of the viral haemorrhagic pneumonia of the wild rabbit on the diet and breeding success of the Golden Eagle *Aquila chrysaetos* (L.). Rev Ecol Terre Vie 48: 323-329.
3. García-Dios IS (2006) Dieta del aguililla calzada en el sur de Ávila: importancia de los paseriformes. Ardeola 53: 39-54.
4. Gil-Sánchez JM, Moleón M, Otero M, Bautista J (2004) A nine-year study of successful breeding in a Bonelli’s eagle population in southeast Spain: a basis for conservation. Biol Conserv 118: 685-694.
5. Gil-Sánchez JM, Ballesteros-Duperón E, Bueno-Segura J (2006) Feeding ecology of the Iberian lynx *Lynx pardinus* in eastern Sierra Morena (Southern Spain). Acta Theriol 51: 85-90.
6. Lozano J, Moleón M, Virgós E (2006) Biogeographical patterns in the diet of the wildcat, Felis silvestres Schreber 1775, in Eurasia: factors affecting the trophic diversity. J Biogeogr 33: 1076-1085.
7. Mañosa S (1994) Goshawk diet in a Mediterranean area of northeastern Spain. J Raptor Res 28: 84-92.
8. Martínez JE, Calvo JF (2005) Prey partitioning between mates in breeding booted eagles (*Hieraaetus pennatus*). J Raptor Res 39: 159-163.
9. Moleón M (2007) El estudio del impacto de los predadores sobre las presas cinegéticas: un intento de compatibilizar caza y conservación. In: Barea-Azcón JM, Moleón M, Travesí R, Ballesteros-Duperón E, Luzón JM, et al., editors. Biodiversidad y Conservación de Fauna y Flora en Ambientes Mediterráneos. Granada: Sociedad Granatense de Historia Natural. pp. 743-794.
10. Moleón M (2007) Interacciones ecológicas entre depredadores y presas: águilas perdiceras, conejos y perdices. PhD thesis. Granada: Universidad de Granada. 162p.
11. Zuberogoitia I, Martínez JE, Martínez JA, Zabala J, Calvo JF, et al. (2006) Influence of management practices on nest site habitat selection, breeding and diet of the common buzard *Buteo buteo* in two different areas of Spain. Ardeola 53: 83-98.
